# Schnitzler’s metastasis from gastric carcinoma: A multimodality imaging case report

**DOI:** 10.4102/sajr.v30i1.3567

**Published:** 2026-10-29

**Authors:** H. Nadeem Ur Rahman, S. Mac Win

**Affiliations:** 1 Department of Radiodiagnosis, Chettinad Academy of Research and Education, Chennai, India; 2 Department of Radiodiagnosis, Christian Medical College, Vellore, India

**Keywords:** Schnitzler’s metastasis, gastric carcinoma, rectal metastasis, MRI rectum, CT abdomen, multimodality imaging, rectal wall thickening, submucosal infiltration

## Abstract

Schnitzler’s metastasis – haematogenous or lymphatic spread of gastric carcinoma to the rectal wall – is a rare entity that radiologically closely mimics primary rectal adenocarcinoma, creating a risk of misdiagnosis and inappropriate treatment. A 57-year-old woman presented with progressive constipation, tenesmus and rectal discomfort. Contrast-enhanced CT demonstrated circumferential gastric wall thickening, alongside circumferential rectal wall thickening with avid mucosal enhancement. Pelvic MRI revealed submucosal rectal wall thickening with preserved mesorectal fat planes and no significant diffusion restriction. Endoscopic biopsy confirmed metastatic adenocarcinoma of gastric origin.

**Contribution:**

Schnitzler’s metastasis should be considered in any patient with upper gastrointestinal malignancy presenting with rectal symptoms. Combined CT and pelvic MRI can assist in accurate characterisation, extent assessment and identification of the gastric primary.

## Introduction

### Background

Gastric carcinoma is the fifth most common malignancy and the fourth leading cause of cancer-related mortality worldwide.^1^ Metastatic dissemination to the rectum – eponymously termed Schnitzler’s metastasis after Ludwig Schnitzler, who first described perirectal infiltration from gastric cancer in 1939 – is exceedingly rare.^2,3^ A systematic review of 41 published cases identified a median patient age of 59 years, with poorly differentiated adenocarcinoma predominating (86.1%), including 38.9% signet-ring cell carcinoma.^4^ Clinically, constipation, tenesmus and rectal discharge closely mimic primary rectal carcinoma; and superficial endoscopic biopsies frequently return false-negative results owing to the predominantly submucosal metastatic infiltrate.^4,5^ This report describes a similar case in a 57-year-old woman, highlighting the multimodality CT and MRI features that enable accurate pre-biopsy characterisation.

## Case presentation

A 57-year-old woman with no significant past medical history, no family history of malignancy, no history of smoking or alcohol use, and no prior gastrointestinal illness, presented with 6 weeks of worsening constipation, tenesmus and blood-streaked stools, accompanied by a 4 kg unintentional weight loss and early satiety. Digital rectal examination revealed firm, indurated circumferential narrowing approximately 6 cm from the anal verge. Laboratory investigations indicated microcytic anaemia (haemoglobin 9.8 g/dL), elevated carcinoembryonic antigen (CEA) at 18.4 ng/mL and cancer antigen 19-9 (CA 19-9) at 112 U/ml. Upper and lower gastrointestinal endoscopies were subsequently arranged. Portal venous phase contrast-enhanced CT (1.25 mm reconstructions), performed within the same week of presentation, demonstrated irregular, circumferential wall thickening in the gastric body, up to 30 mm, with loss of mural stratification, perigastric fat stranding and enlarged lesser-curve lymph nodes (largest 15 mm in short-axis), consistent with a Borrmann type III/IV gastric carcinoma (Figure 1). The mid and upper rectum demonstrated circumferential wall thickening spanning approximately 7 cm craniocaudally, beginning 5 cm above the anorectal junction, with a maximal wall thickness of 14 mm and avid layered portal venous phase enhancement. No discrete perirectal nodes, hepatic lesions or peritoneal deposits were identified.

**FIGURE 1:**
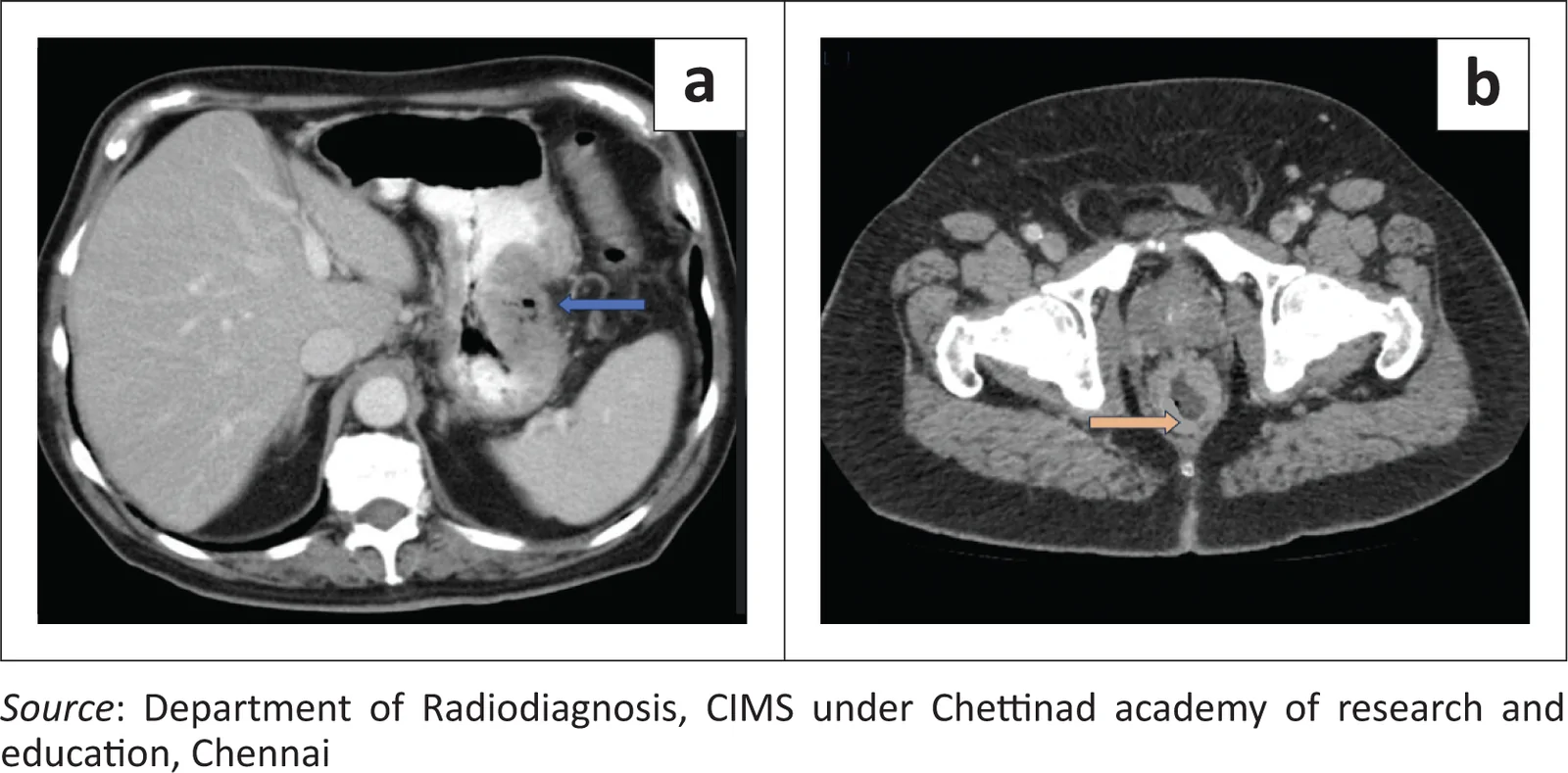
Contrast-enhanced CT abdomen: (a) Axial plane at the level of the stomach – Irregular circumferential wall thickening of the gastric body along the greater curvature (blue arrow) with heterogeneous enhancement and perigastric fat stranding. (b) Axial plane at the level of rectum – Circumferential mural thickening (orange arrow) causing mild luminal narrowing.

The CT detection of incidental, non-mass-like rectal wall abnormality prompted a dedicated pelvic MRI. Imaging parameters, (1.5-T magnet; axial, sagittal and coronal oblique T2-weighted turbo spin-echo imaging [TR 4500 ms – 6000 ms, TE 90 ms – 110 ms]; axial diffusion-weighted imaging with b-values of 0 s/mm^2^, 800 s/mm^2^ and 1000 s/mm^2^ [TR 4000 ms – 5000 ms, TE 60 ms – 70 ms] and corresponding ADC mapping; and axial fat-suppressed post-contrast T1-weighted imaging [TR 550 ms – 650 ms, TE 10 ms –15 ms] following intravenous gadodiamide [Omniscan] 0.1 mmol/kg). Scans demonstrated circumferential intermediate-to-low signal intensity thickening of the mid and upper rectal wall, predominantly involving the submucosa and muscularis propria with relative preservation of the overlying mucosal surface – a pattern more consistent with secondary submucosal infiltration than a primary mucosal neoplasm (Figure 2). The mesorectal fat plane and fascia were intact with no mesorectal lymphadenopathy. Diffusion-weighted imaging showed no significant restriction, with a normal ADC map – a finding attributable to the desmoplastic fibrous stroma of the metastatic deposit (Figure 2c). Post-contrast T1-weighted imaging demonstrated avid layered ‘target sign’ enhancement: a brightly enhancing inner mucosal layer and outer muscularis propria framing a hypo-enhancing submucosal band, suggestive of metastatic submucosal infiltration (Figure 2d).

**FIGURE 2:**
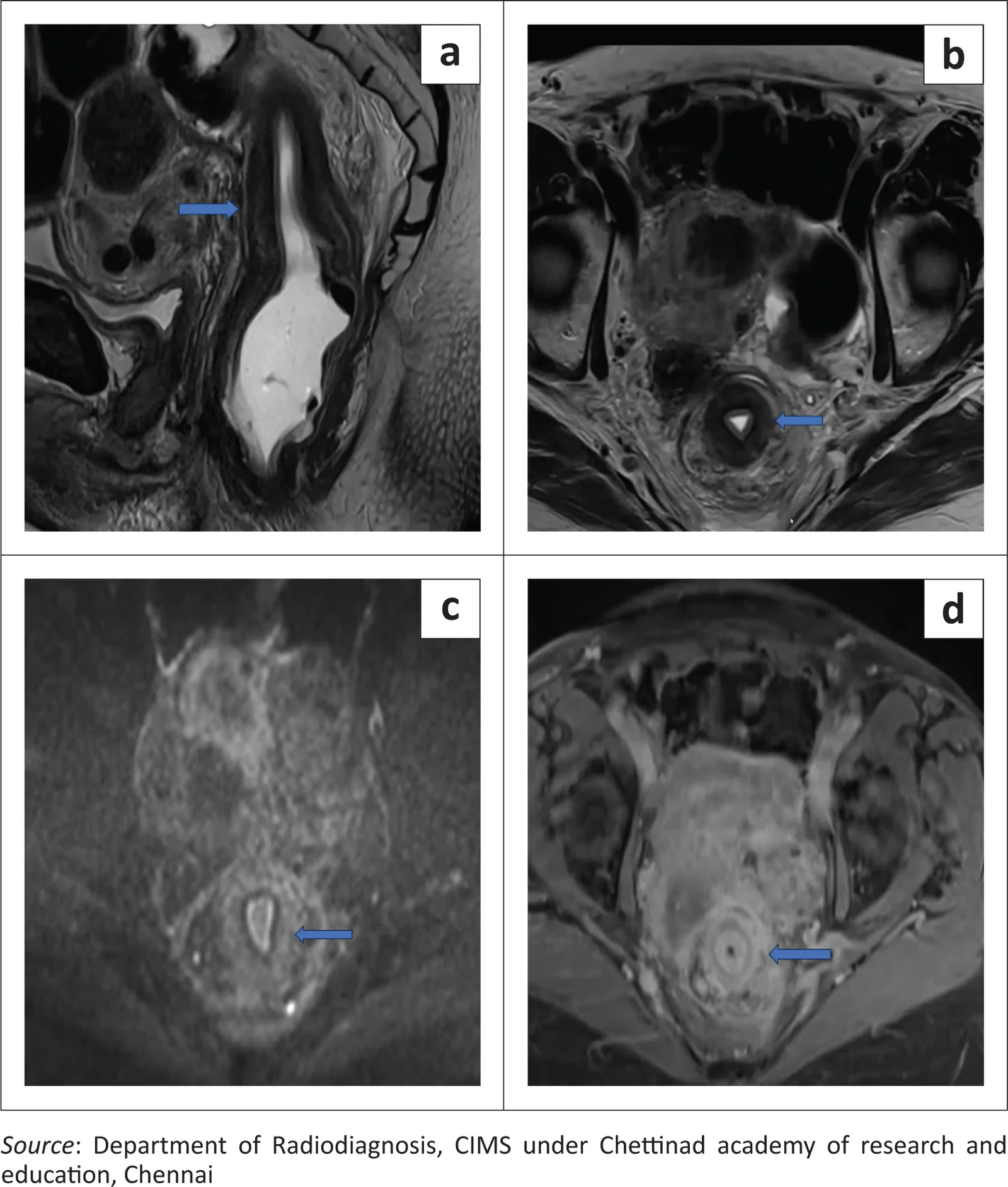
MRI pelvis: (a) Sagittal T2-weighted imaging shows the craniocaudal extent of the circumferential rectal wall thickening (arrow), which spans approximately 7 cm, with associated luminal stenosis. (b) Axial T2-weighted imaging demonstrates circumferential T2 hypointense mural thickening of the mid and upper rectal wall (arrow), predominantly involving the submucosal and muscularis propria layers. The mesorectal fat plane and mesorectal fascia are intact. (c) Axial ADC. The thickened rectal wall (blue arrow) showed no significant diffusion restriction with no signal drop on ADC. (d) Axial fat-suppressed post-contrast T1-weighted MRI – avid, layered ‘target sign’ enhancement of the thickened rectal wall (arrow), with a prominent enhancing inner mucosal layer and enhancing outer muscularis propria separated by a hypo-enhancing submucosal layer.

Upper gastrointestinal endoscopy, performed 2 weeks after presentation, identified an ulcero-proliferative gastric body mass; biopsy confirmed poorly differentiated adenocarcinoma with signet-ring cell features. Colonoscopy revealed oedematous, circumferentially narrowed mid-rectal mucosa with a deceptively preserved surface; superficial biopsies returned only non-specific chronic inflammation, consistent with the submucosal biology of Schnitzler’s metastasis. Deep 18-gauge coaxial core biopsy of the rectal wall, performed a week later, yielded the diagnosis: poorly differentiated metastatic adenocarcinoma with signet-ring cell features. Immunohistochemistry demonstrated Cytokeratin 7(CK7)-positive, MUC5AC-positive, Cytokeratin 20(CK20)-negative, and Human Epidermal Growth Factor Receptor 2 (HER2) score 2+ – a profile more typically associated with gastric than colorectal origin (Figure 3). The converse CK7-negative and CK20-positive phenotype predominates in colorectal adenocarcinoma.^6^ Given this immunoprofile, together with the unequivocal gastric primary on endoscopy and imaging, confirmatory HER2 in situ hybridisation/Fluorescence in situ hybridization (ISH/FISH) for the equivocal 2+ score was not pursued, as it was not anticipated to alter the palliative-intent management plan.

**FIGURE 3:**
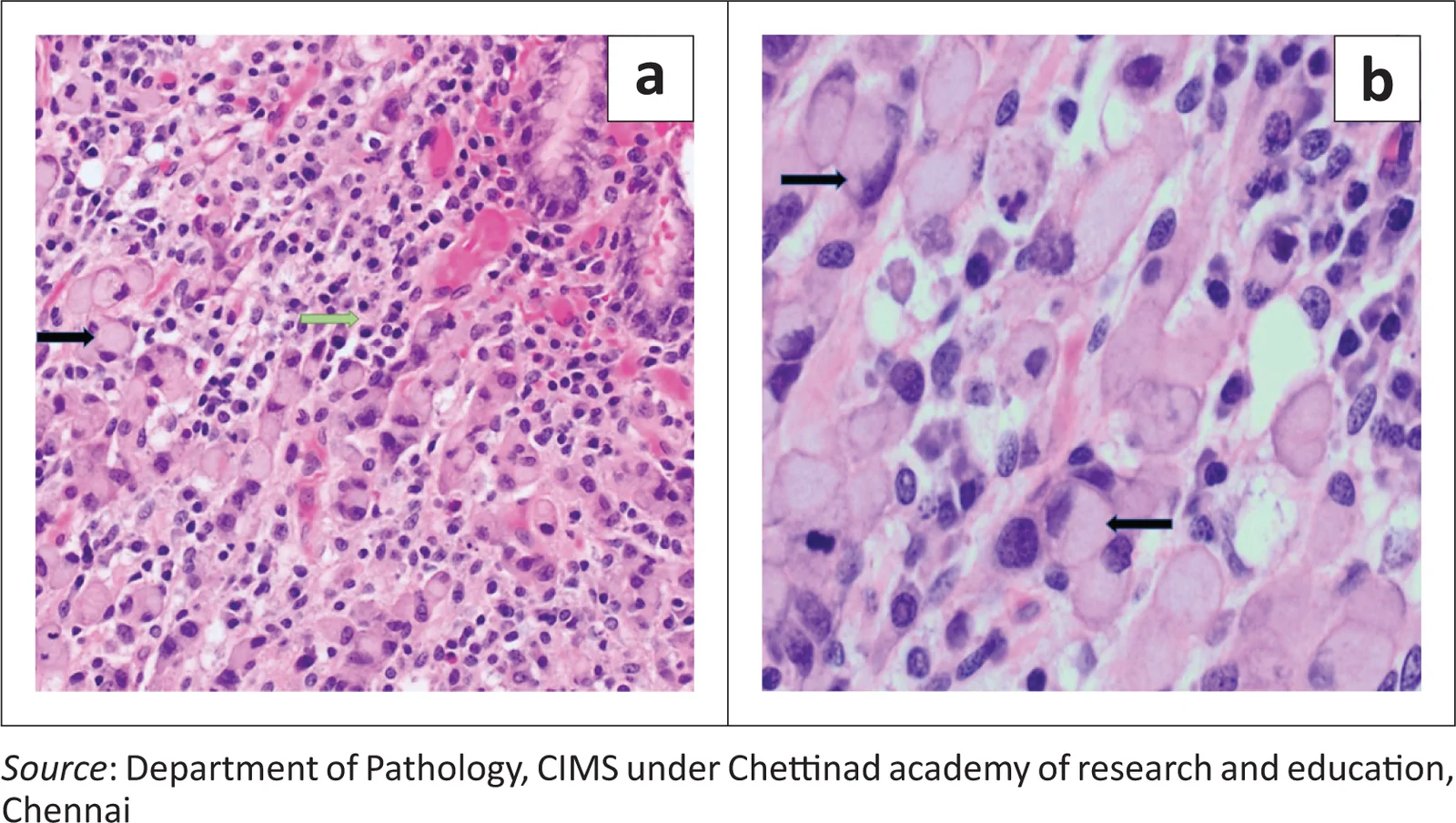
Photomicrograph from the deep rectal core biopsy, haematoxylin and eosin stain: (a) Low-power view demonstrating sporadic diffuse gastric-type adenocarcinoma metastatic to the rectal wall, arising on a background of chronic inflammation. The tissue shows architectural distortion with scattered signet ring cells (black arrow), identified and dispersed amid the inflammatory stroma (green arrow), consistent with poorly differentiated diffuse-type adenocarcinoma. (b) High-power view of the same biopsy specimen showing individual and clustered signet ring cells (black arrows) characterised by abundant intracytoplasmic mucin vacuoles, eccentrically displacing the nucleus to the cell periphery, imparting the classical signet ring morphology. No gland formation is identified.

Multidisciplinary tumour board review, held the following week, confirmed synchronous stage IV disease (cT3N2M1), precluding curative-intent surgery. Palliative folinic acid, fluorouracil and oxaliplatin (FOLFOX) chemotherapy was initiated alongside nutritional optimisation and early palliative care involvement. Progressive rectal stenosis subsequently necessitated a defunctioning loop colostomy to maintain bowel function and quality of life. Response to chemotherapy was assessed with contrast-enhanced CT after every three cycles of FOLFOX in accordance with RECIST 1.1, alongside serial CEA and CA 19-9 monitoring and clinical assessment of bowel symptoms. Following the defunctioning colostomy, the patient reported symptomatic relief of tenesmus and obstructive symptoms and remains under continued oncological follow-up.

## Discussion

Schnitzler’s metastasis constitutes a significant diagnostic pitfall: its submucosal biology produces a macroscopic endoscopic appearance of preserved but oedematous mucosa, leading to a high rate of false-negative superficial biopsies.^5^ Pathogenesis is attributed to retrograde lymphatic dissemination via mesorectal channels, haematogenous spread via the portal or systemic circulation, and transperitoneal seeding with subsequent submucosal infiltration.^3,7^

In the presented case, CT served a dual function: identifying the gastric primary while simultaneously flagging the rectal abnormality. The layered enhancement pattern of the thickened rectal wall on portal venous phase imaging provided an early indication against a primary rectal tumour, as prior reports have noted that metastatic submucosal infiltration tends to produce this stratified appearance, contrasting with the homogeneous or nodular enhancement more typical of primary rectal adenocarcinoma.^4,8^

MRI provided greater diagnostic specificity compared to CT. The intermediate-to-low T2 signal concentrated in the submucosa and muscularis propria – with an intact mucosal surface – differs fundamentally from the mass-like T2 signal of primary rectal adenocarcinoma, which typically disrupts mucosa early and invades mesorectal fat. T2 hypo-intensity in this setting reflects the desmoplastic fibrous stroma that can be induced by metastatic signet-ring cell carcinoma.^3,9^ Preserved mesorectal fat planes further supported a non-primary rectal tumour. The absent diffusion restriction in this case merits specific comment. Unlike most rectal malignancies, the thickened wall here showed no meaningful restriction and a normal ADC map, leading to initial diagnostic uncertainty. This finding aligns with the histological composition of Schnitzler’s metastasis: tumour cells dispersed within dense fibrous stroma, where the stromal component dominates the tissue architecture and permits freer water diffusion.^10^ Radiologists must bear this in mind: a normal DWI in a patient with known gastric cancer and suspicious rectal thickening on other sequences warrants deep biopsy, not reassurance.^11^ The ‘target sign’ on post-contrast T1 imaging – brisk mucosal and muscularis propria enhancement framing a hypo-enhancing submucosal zone – was among the most informative observations. This appearance has been previously documented in Schnitzler’s metastasis and reflects the zonal architecture of submucosal tumour infiltration,^12^ distinguishing it from the uniform enhancement of primary rectal carcinoma and the serosal involvement of peritoneal carcinomatosis.

The combination of a gastric primary on CT, submucosal T2 signal pattern on MRI, a layered enhancement pattern and absence of an intraluminal mass should prompt deep core biopsy with immunohistochemical profiling rather than radical surgery.^5,13^ Circumferential rectal wall thickening in a patient with a known gastric primary raises several imaging differentials, summarised in Table 1.

**TABLE 1:** Imaging differential diagnosis of rectal wall thickening.

Conditions and/or disease	Distinguishing imaging features
Schnitzler’s metastasis (this case)	Submucosal, mucosa-sparing T2 hypointense thickening; layered ‘target sign’ enhancement; no diffusion restriction; known gastric primary
Primary rectal adenocarcinoma	Mucosal-origin mass disrupting the mucosal surface early; invades mesorectal fat; nodular or heterogeneous enhancement; typically restricted diffusion
Rectal lymphoma	Bulky, homogeneously enhancing mass or marked circumferential thickening with preserved perirectal fat planes; no desmoplastic reaction; lacks layered enhancement^14^
Rectal linitis plastica (primary or 2° to gastric, breast cancer)	Concentric ‘target sign’ with desmoplastic wall thickening, often indistinguishable from Schnitzler’s metastasis on imaging alone; diagnosis relies on the identified primary and clinical context^15^
Other metastatic disease (e.g. ovarian, etc)	Similar infiltrative pattern to linitis plastica; distinguished chiefly by a known extra-rectal primary
Inflammatory rectal disorders (e.g. proctitis)	Stratified mural enhancement can mimic a target sign, but accompanied by perirectal fat stranding, engorged mesorectal vessels (‘comb sign’), and inflammatory rather than obstructive symptoms

Management is predominantly palliative. While surgical resection has been reported for obstructive symptoms in selected cases,^9,16^ systemic chemotherapy is the mainstay for most patients. Median overall survival from gastric cancer diagnosis has been reported at 72 months, with survival from diagnosis of rectal metastasis considerably shorter at 16 months,^4^ underscoring the importance of early recognition and prompt initiation of appropriate therapy.

## Conclusion

Schnitzler’s metastasis is a rare but clinically significant pattern of gastric carcinoma spread that can masquerade as primary rectal cancer. Recognising its multimodality imaging features – circumferential submucosal rectal wall thickening with layered CT and MRI enhancement, preserved mucosa and absence of an intraluminal mass – can help radiologists identify this entity in patients with a known upper gastrointestinal primary. Diffusion restriction may be absent in cases with a predominantly desmoplastic infiltrate, and its absence should not preclude further investigation when other imaging features are suspicious. Early radiological recognition prevents misdiagnosis, avoids inappropriate radical surgery and enables timely staging, palliative systemic therapy and patient counselling.
